# The prevalence and risk factor control associated with noncommunicable diseases in China, Japan, and Korea

**DOI:** 10.1016/j.je.2016.12.019

**Published:** 2017-06-13

**Authors:** Defu Ma, Hiromichi Sakai, Chihiro Wakabayashi, Jong-Sook Kwon, Yoonna Lee, Shuo Liu, Qiaoqin Wan, Kumiko Sasao, Kanade Ito, Ken Nishihara, Peiyu Wang

**Affiliations:** a School of Public Health, Peking University Health Science Center, Beijing, China; b Saitama Prefectural University, Saitama, Japan; c Shingu College, Seongnam, South Korea; d Beijing Cancer Hospital, Peking University, Beijing, China; e School of Nursing, Peking University Health Science Center, Beijing, China

**Keywords:** Noncommunicable disease, Risk control, Prevalence

## Abstract

**Background:**

Noncommunicable disease (NCD) has become the leading cause of mortality and disease burden worldwide.

**Methods:**

A cross-sectional survey was carried out to investigate the prevalence of NCDs and risk factor control on dietary behaviors and dietary intake in China, Japan, and Korea.

**Results:**

There were significant differences among the three countries on the prevalence of hypertension (24.5% in China, 17.6% in Korea, and 15.2% in Japan), diabetes (8.9% in China, 5.7% in Korea, and 4.8% in Japan), hyperlipidemia (13.1% in China, 9.2% in Korea, and 6.9% in Japan), and angina pectoris (3.6% in China, 1.7% in Korea, and 1.5% in Japan). The prevalence rate of hypertension, diabetes, hyperlipidemia, and angina pectoris was highest in China and lowest in Japan. However, 82.2%, 48.4%, and 64.4% of Chinese, Koreans, and Japanese presented good dietary behavior, respectively. Multivariable logistic regression analysis found that sex, age, and marital status were predictors of good dietary behavior. In addition, in comparison with subjects without hypertension, diabetes, or hyperlipidemia, subjects with hypertension, diabetes, or hyperlipidemia significantly improved their dietary behaviors and controlled their intake of salt, sugar, and oil.

**Conclusions:**

The prevalence of NCDs and trends in major modifiable risk factor control in China, Korea, and Japan remain troubling. Public efforts to introduce healthy lifestyle changes and systematic NCDs prevention programs are necessary to reduce the epidemic of NCDs in these three Asian countries.

## Highlights

• The prevalences of hypertension, diabetes, and hyperlipidemia were highest in China.
• The prevalences of hypertension, diabetes, and hyperlipidemia were lowest in Japan.
• Some dietary behaviors improved hypertension, diabetes, and hyperlipidemia.
• These dietary behaviors differed by sex, age, and marital status.

## Introduction

A noncommunicable disease (NCD) is a medical condition that is noninfectious and nontransmissible among people. NCD is one of the leading causes of mortality and disease burden worldwide. A total of 34.5 million deaths (65% of total deaths) globally were caused by NCDs in 2010, which showed a significant increase from 1990.^1^ The morbidity and mortality rates of NCDs in China, Japan, and Korea have increased because of increasing fat and protein intake and sedentary lifestyle.^2^, ^3^ According to the national data, 80% of deaths and 70% of the total disease burden in China in 2005 were caused by NCDs.^4^ Using the mortality and national health insurance claim data in Korea, researchers found that, of all causes of mortality, the proportion of mortality from the major NCDs was 39.4% in 1983 and increased to 56.0% in 2011.^5^ In Japan, the leading causes of death are malignant neoplasm, heart disease, and cerebrovascular disease, which accounted for more than 50% of the total deaths in 2009.^6^

To reduce the burden of NCDs, comprehensive approaches across the entire disease spectrum are needed, from health promotion, primary prevention, high-risk group screening, and early diagnosis to better treatment and rehabilitation. Among these comprehensive approaches, risk factor modification is an efficient and proven strategy in reducing NCD burden.^7^ According to the World Health Organization (WHO) report, tobacco and alcohol use, physical inactivity, unhealthy dietary behavior, and dietary intake status have been identified as major determinants of NCDs.^8^ However, “nutrition transition” from traditional vegetable dietary pattern to unhealthy dietary intake, such as processed food, fast food, and fried food, is the decisive factor in the rapid growth of NCD burden for Chinese, Japanese, and Koreans.^9^ Recently, most public health activities have targeted modifiable risk factors in reducing the burden of NCDs because the prevention and treatment of major modifiable risk factors have been proven to be effective in reducing mortality caused by NCDs. Over the past few decades, we observed some significant achievements in risk factor modifications among Chinese, Japanese, and Koreans, such as improved dietary behaviors and dietary intake status.^7^

In spite of the many reports on the prevalence of NCDs and risk factor control among Chinese, Japanese, and Koreans, direct comparison of the prevalence and risk factor control between Chinese, Japanese, and Koreans, has never been investigated under the same study protocol. In the present research, the prevalence of NCDs and risk factor control, including dietary behaviors and dietary intake status among Chinese, Japanese, and Koreans, were compared by using similar study protocols.

## Methods

### Sampling

This study is cooperative research that is performed by Peking University in China, Saitama Prefectural University in Japan, and Shingu College in Korea. In this study, a cross-sectional survey was performed to investigate the prevalence of NCDs and risk factor control on dietary behaviors and dietary intake in four cities, including Beijing in China, with a population of about 20 million; Matsumoto and Koshigaya in Japan, with populations of about 240,000, and 330,000, respectively; and Seongnam in Korea, with a population of about 1 million. Beijing is the capital of China. Matsumoto, Koshigaya, and Seongnam are average cities in Japan and Korea. A multistage stratified random sampling was used in the study. These four cities were selected using purposive sampling in each country, and three communities in different districts were randomly selected in China and Korea. Then, face-to-face interviews were conducted to answer the questionnaire in China and Korea. In Japan, 1500 subjects were randomly selected from the registered permanent residents in Matsumoto and Koshigaya, and self-administered questionnaires were mailed to collect information from registered permanent residents. Participation was noncompulsory, and the respondents were asked to mail back the questionnaires. The study was conducted according to the guidelines in the Declaration of Helsinki. All of the procedures involving human subjects were approved by the Medical Ethics Research Board of Peking University, Saitama Prefectural University, and Shingu College. A written informed consent was obtained in the study.

### Questionnaire interviews

The prevalence of four major NCDs, including hypertension, diabetes, hyperlipidemia, and angina pectoris, was investigated in this study. The health outcomes were obtained using a questionnaire. Demographic information, such as age, sex, household composition, education, marital status, and employment status, were obtained. In this study, marital status was stratified to four groups: single, married, divorced, or widowed. The educational status included four levels: less than primary schools, middle school, high school, junior college and technical secondary school, or college and graduate school. In this study, weight and height were self-reported. All subjects were stratified according to the WHO body mass index (BMI) classification: BMI less than 18.5 kg/m^2^ was classified as underweight, BMI 18.5 kg/m^2^ to 24.9 kg/m^2^ was classified as normal weight, BMI 25.0 kg/m^2^ to 29.9 kg/m^2^ was classified as overweight, and BMI 30.0 kg/m^2^ or greater was classified as obese.

Nine questions on dietary behaviors were asked: 1) “Do you control salt intake?”; 2) “Do you control sugar intake?”; 3) “Do you control oil intake?”; 4) “Do you control the intake of food with additives?”; 5) “Do you control the intake of too much energy?”; 6) “Do you eat on time?”; 7) “Do you care about nutrition balance?”; 8) “Do you drink adequate water?”; and 9) “Do you buy food according to the nutrition label?”. Moreover, the dietary behaviors were quantified by marks, and each good dietary behavior was given one mark. A participant who obtained more than five marks was classified as having good dietary behavior. In addition, a food frequency questionnaire was used to investigate the intake status of 12 kinds of food: fruits; vegetables; milk and dairy; beans, tofu and soymilk; seaweed; fish; meat; processed meat; instant noodles; breakfast; eating out; and fast food. The intake frequencies included four classes: never, 1–2 days each week, 3–4 days each week, or 5 or more days each week.

The questionnaire was produced in Japan, and it was translated to Chinese and Korean by Chinese and Korean researchers, respectively. In addition, the questionnaire in Chinese and Korean was back-translated to Japanese to determine its consistency. The validation of each questionnaire version was evaluated using a pilot study in each country.

### Data analysis

The differences in the proportion of demographic variables, dietary behaviors, and dietary intake status among three countries were analyzed using the chi-square test. Multivariable logistic regression analysis was used to clarify the predictors for good dietary behavior. The mean score of dietary behavior was analyzed using analysis of variance. Chi-square test was performed to analyze the differences in the prevalence of hypertension, diabetes, hyperlipidemia, and angina pectoris among the three countries. In addition, chi-square test was used to determine the differences on dietary behavior and dietary intake status between subjects with noncommunicable disease and subjects without noncommunicable disease. *P* values were two-tailed, with *P* < 0.05 being considered statistically significant. Statistical analyses were performed using SPSS 20.0 (SPSS Inc., Chicago, IL, USA).

## Results

In the present research, 1742, 905, and 3000 subjects were investigated and 1742, 905, and 1667 valid questionnaires were obtained in China, Korea, and Japan, respectively. The response rate was 100%, 100%, and 55.6% in China, Korea, and Japan, respectively. There were significant differences in the prevalence of hypertension (24.5% in China, 17.6% in Korea, and 15.2% in Japan, *P* = 0.001), diabetes (8.9% in China, 5.7% in Korea, and 4.8% in Japan, *P* = 0.001), hyperlipidemia (13.1% in China, 9.2% in Korea, and 6.9% in Japan, *P* = 0.001), and angina pectoris (3.6% in China, 1.7% in Korea, and 1.5% in Japan, *P* = 0.001) among the three countries. The prevalence rate of hypertension, diabetes, hyperlipidemia, and angina pectoris was highest in China and lowest in Japan. Demographic characteristics of the subjects in the three countries are summarized in Table 1. The distributions of sex, age, marital status, education, and BMI were significantly different among the three countries (*P* = 0.001).

**Table 1:** Demographic characteristics of the subjects in China, Korea, and Japan.

Indices	China N (%)	Korea N (%)	Japan N (%)	*P*
**Sex**				
Male	691 (39.8)	322 (35.6)	761 (45.7)	<0.01
Female	1047 (60.2)	583 (64.4)	906 (54.3)	
**Age, years**				
20–29	287 (16.5)	220 (24.3)	207 (12.5)	<0.01
30–39	307 (17.6)	176 (19.4)	316 (19.0)	
40–49	286 (16.4)	143 (15.8)	346 (20.9)	
50–59	418 (24.0)	189 (20.9)	326 (19.7)	
>60	444 (25.5)	177 (19.6)	464 (28.0)	
**Marriage**				
Single	212 (12.2)	266 (29.4)	327 (19.7)	<0.01
Married	1464 (84.0)	574 (63.5)	1212 (73.0)	
Divorced	29 (1.7)	22 (2.4)	87 (5.2)	
Widowed	37 (2.1)	42 (4.6)	34 (2.0)	
**Education**				
Less than primary schools	177 (10.2)	69 (7.6)	2 (0.1)	<0.01
Middle School	564 (32.5)	96 (10.6)	138 (8.3)	
High School	307 (17.7)	237 (26.2)	631 (38.1)	
Junior College, Technical Secondary School	379 (21.8)	191 (21.2)	451 (27.3)	
College, Graduate School	310 (17.8)	310 (34.3)	432 (26.1)	
**BMI, kg/m**^**2**^				
<18.5	74 (4.3)	41 (4.6)	146 (9.0)	<0.01
18.5–24.9	947 (54.4)	659 (73.5)	1151 (70.9)	
25–30	607 (34.9)	170 (19.0)	271 (16.6)	
>30	112 (6.4)	26 (2.9)	58 (3.6)	

BMI, body mass index.

*P* values were calculated using the Chi-square test.

Table 2 shows the dietary behavior control status in China, Korea, and Japan. There were significant differences on the control of salt, sugar, oil, and calories among participants in China, Korea, and Japan. More than 70% of Chinese tried their best to control the intake of salt, sugar, oil, and calories. However, only 35.3% of Koreans tried their best to control the intake of calories. For the dietary behaviors of food additive control, eating on time, paying attention to nutrition balance and labels, and taking adequate amounts of water, significant differences among participants in China, Korea, and Japan were also observed. Most of the subjects tried their best to follow good dietary behaviors. However, most subjects did not pay attention to nutrition labels. Only 44.1% of Chinese, 33% of Koreans, and 28% of Japanese paid attention to nutrition labels. To explore the predictors of good dietary behavior, the dietary behaviors were quantified by marks, and each good dietary behavior was given one mark. Significant differences were observed among the three countries, and the mean scores were 6.74, 4.37, and 5.32 in China, Korea, and Japan, respectively (*P* = 0.001). Subjects who obtained more than 5 marks were classified as showing good dietary behavior, and the prevalence rates of good dietary behavior were 82.2%, 48.4%, and 64.4% in China, Korea, and Japan, respectively (*P* = 0.001). Multivariable logistic regression analysis found that sex, age, and marital status were the predictors of good dietary behavior, after adjustment for BMI and education level. Female, married, and old-aged subjects had better dietary behavior than other subjects (Table 3).

**Table 2:** The differences of dietary behaviors in China, Korea, and Japan.

	China N (%)	Korea N (%)	Japan N (%)	*P*
**Salt-control**				
Yes	1304 (74.9)	463 (51.2)	1005 (60.5)	<0.01
No	437 (25.1)	441 (48.8)	656 (39.5)	
**Sugar-control**				
Yes	1261 (72.4)	434 (48.0)	985 (59.4)	<0.01
No	481 (27.6)	470 (52.0)	673 (40.6)	
**Oil-control**				
Yes	1368 (78.6)	524 (57.9)	1085 (65.3)	<0.01
No	373 (21.4)	381 (42.1)	577 (34.7)	
**Food additives-control**				
Yes	1291 (74.3)	461 (51.1)	804 (48.5)	<0.01
No	447 (25.7)	441 (48.9)	854 (51.5)	
**Eat on time**				
Yes	1586 (91.9)	443 (49.0)	1012 (61.0)	<0.01
No	154 (8.9)	461 (51.0)	646 (39.0)	
**Calorie-control**				
Yes	1300 (74.7)	319 (35.3)	1106 (66.7)	<0.01
No	440 (25.3)	585 (64.7)	552 (33.3)	
**Pay attention to the nutrition balance**				
Yes	1327 (76.2)	409 (45.2)	1210 (72.9)	<0.01
No	414 (23.8)	496 (54.8)	449 (27.1)	
**Drink adequate water**				
Yes	1528 (87.8)	602 (66.5)	1169 (70.5)	<0.01
No	214 (12.3)	303 (33.5)	490 (29.5)	
**Pay attention to the nutrition label**				
Yes	767 (44.1)	229 (33.0)	465 (28.0)	<0.01
No	973 (55.9)	606 (67.0)	1194 (72.0)	

*P* values were calculated by Chi-square test.

**Table 3:** The predictors for good dietary behavior in China, Korea, and Japan.

	OR (95% CI) in China	OR (95% CI) in Korea	OR (95% CI) in Japan
**Sex**			
Male	Reference	Reference	Reference
Female	2.99 (2.28, 3.91)^∗^	1.50 (1.09, 2.06)^∗^	3.27 (2.55, 4.20)^∗^
**Age, years**			
20–29	Reference	Reference	Reference
30–39	0.93 (0.57, 1.53)	1.34 (0.76, 2.37)	1.55 (1.03, 2.33)
40–49	1.03 (0.6, 1.78)	1.56 (0.82, 2.96)	1.84 (1.19, 2.83)*
50–59	1.86 (1.06, 3.29)*	2.90 (1.46, 5.77)*	3.0 (1.91, 4.71)*
>60	2.33 (1.27, 4.28)*	6.11 (2.8, 13.32)*	6.94 (4.27, 11.3)*
**Marriage**			
Single	0.53 (0.32, 0.87)^∗^	0.55 (0.32, 0.94)^∗^	0.94 (0.68, 1.30)
Married	Reference	Reference	Reference
Divorced	0.52 (0.21, 1.31)	0.53 (0.21, 1.33)	0.81 (0.49, 1.33)
Widowed	0.72 (0.27, 1.95)	1.01 (0.48, 2.14)	0.77 (0.30, 2.02)
**Education**			
Less than primary schools	Reference	Reference	Reference
Middle School	1.29 (0.74, 2.23)	0.90 (0.44, 1.83)	1.29 (0.74, 2.23)
High School	1.12 (0.62, 2.03)	0.99 (0.51, 1.94)	1.12 (0.62, 2.03)
Technical Secondary School	1.26 (0.69, 2.29)	1.37 (0.64, 2.89)	1.26 (0.69, 2.29)
College, Graduate School	1.81 (0.95, 3.42)	1.45 (0.71, 2.99)	1.81 (0.95, 3.42)
**BMI. kg/m**^**2**^			
<18.5	Reference	Reference	Reference
18.5–24.9	1.59 (0.89, 2.84)	0.71 (0.36, 1.40)	1.15 (0.77, 1.71)
25–30	1.37 (0.74, 2.54)	0.62 (0.29, 1.33)	0.71 (0.44, 1.14)
>30	1.13 (0.51, 2.48)	0.55 (0.18, 1.66)	0.80 (0.40, 1.60)
**Hypertension**			
Yes	Reference	Reference	Reference
No	0.75 (0.51, 1.10)	0.88 (0.56, 1.38)	0.64 (0.44, 0.94)^∗^
**Diabetes**			
Yes	Reference	Reference	Reference
No	0.65 (0.36, 1.18)	1.05 (0.55, 2.01)	1.39 (0.79, 2.43)
**Hyperlipidemia**			
Yes	Reference	Reference	Reference
No	1.26 (0.82, 1.95)	1.00 (0.59, 1.71)	0.73 (0.44, 1.20)
**Angor pectoris**			
Yes	Reference	Reference	Reference
No	1.76 (0.87, 3.56)	0.81 (0.25, 2.57)	0.90 (0.35, 2.37)

BMI, body mass index; CI, confidence interval; OR, odds ratio.

Adjusted by body mass index and education.

* *P* < 0.05.

The intake of 12 kinds of food is presented in Table 4. Most of the subjects ate fruit at least once a week, but more than 20% of Japanese never ate fruit. More than 80% of Chinese ate vegetables every day, whereas only 27.9% of Koreans ate vegetables every day. About 29.7% of Chinese never ate dairy food, and 42.4% of Chinese never ate seaweed. For the three countries, about 90% or 80% subjects ate soy food or fish at least once a week. Meat was consumed daily by 24.4%, 6.0%, and 14.1% and processed meat was consumed daily by 2.6%, 3.6%, and 4.7% of Chinese, Korean, and Japanese, respectively. About 50% or 70% of subjects never ate instant noodles or fast food, and only half of Koreans ate breakfast daily. There were 54.7%, 33.2%, and 50.2% of Chinese, Koreans, and Japanese subjects who never ate out, respectively.

**Table 4:** The differences of food intake frequency in China, Korea, and Japan.

Foods	Frequency	China N (%)	Korea N (%)	Japan N (%)	*P*	Foods	Frequency	China N (%)	Korea N (%)	Japan N (%)	*P*
Fruit	Never	151 (8.7)	74 (8.2)	366 (22.2)	<0.01	Meat	Never	165 (9.5)	147 (16.2)	60 (3.6)	<0.01
	1–2 days/week	398 (22.9)	314 (34.7)	614 (37.2)			1–2 days/week	618 (35.8)	511 (56.5)	527 (31.9)	
	3–4 days/week	458 (26.4)	287 (31.7)	322 (19.5)			3–4 days/week	524 (30.3)	193 (21.3)	831 (50.4)	
	≥5 days/week	730 (42.0)	230 (25.4)	350 (21.2)			≥5 days/week	421 (24.4)	54 (6.0)	232 (14.1)	
Vegetable	Never	21 (1.2)	50 (5.5)	21 (1.3)	<0.01	Processed meat	Never	953 (55.2)	490 (54.4)	308 (18.7)	<0.01
	1–2 days/week	109 (6.3)	289 (32.0)	167 (10.1)			1–2 days/week	605 (35.0)	308 (34.2)	914 (55.5)	
	3–4 days/week	153 (8.9)	313 (34.6)	351 (21.2)			3–4 days/week	125 (7.2)	71 (7.9)	347 (21.1)	
	≥5 days/week	1445 (83.6)	252 (27.9)	1114 (67.4)			≥5 days/week	45 (2.6)	32 (3.6)	77 (4.7)	
Milk and dairy	Never	513 (29.7)	177 (19.7)	194 (11.8)	<0.01	Instant noodle	Never	1055 (61.0)	398 (44.2)	986 (59.6)	<0.01
	1–2 days/week	515 (29.8)	290 (32.3)	352 (21.4)			1–2 days/week	495 (28.6)	346 (38.4)	559 (33.8)	
	3–4 days/week	274 (15.9)	239 (26.6)	338 (20.5)			3–4 days/week	97 (5.6)	113 (12.5)	92 (5.6)	
	≥5 days/week	426 (24.7)	191 (21.3)	763 (46.3)			≥5 days/week	82 (4.7)	44 (4.9)	16 (1.0)	
Bean tofu, soymilk	Never	217 (12.5)	95 (10.5)	112 (6.8)	<0.01	Breakfast	Never	85 (4.9)	170 (18.9)	188 (11.4)	<0.01
	1–2 days/week	927 (53.6)	400 (44.4)	595 (36.0)			1–2 days/week	106 (6.2)	110 (12.2)	68 (4.1)	
	3–4 days/week	373 (21.5)	266 (29.5)	506 (30.6)			3–4 days/week	117 (6.8)	135 (15.0)	86 (5.2)	
	≥5 days/week	214 (12.4)	140 (15.5)	440 (26.6)			≥5 days/week	1411 (82.1)	485 (53.9)	1313 (79.3)	
Seaweed	Never	732 (42.4)	182 (20.2)	177 (10.7)	<0.01	Outdoor dining	Never	946 (54.7)	300 (33.2)	832 (50.2)	<0.01
	1–2 days/week	823 (47.7)	479 (53.1)	758 (45.9)			1–2 days/week	329 (19.0)	414 (45.8)	608 (36.7)	
	3–4 days/week	104 (6.0)	181 (20.1)	485 (29.3)			3–4 days/week	171 (9.9)	135 (15.0)	116 (7.0)	
	≥5 days/week	68 (3.9)	60 (6.7)	233 (14.1)			≥5 days/week	284 (16.4)	54 (6.0)	100 (6.0)	
Fish	Never	386 (22.4)	215 (23.9)	110 (6.7)	<0.01	Fast Food	Never	1430 (82.4)	593 (65.6)	1249 (75.3)	<0.01
	1–2 days/week	1116 (64.7)	512 (56.8)	717 (43.4)			1–2 days/week	220 (12.7)	254 (28.1)	394 (23.8)	
	3–4 days/week	150 (8.7)	141 (15.6)	652 (39.4)			3–4 days/week	51 (2.9)	35 (3.9)	10 (0.6)	
	≥5 days/week	72 (4.2)	33 (3.7)	174 (10.5)			≥5 days/week	34 (2.0)	22 (2.4)	5 (0.3)	

*P* values were calculated using the Chi-square test.

The differences on dietary behavior and dietary intake status between subjects with noncommunicable disease and subjects without noncommunicable disease were analyzed. As shown in Table 5, significant differences between subjects with hypertension or diabetes and subjects without hypertension or diabetes on dietary behaviors (except for nutrition balance) were also observed. In comparison with subjects without hyperlipidemia, subjects with hyperlipidemia significantly controlled their intake of salt, sugar, oil, and food additives. Significant differences on food additive-control and eating on time were observed between subjects with angina pectoris and subjects without angina pectoris. Table 6 shows the differences on dietary intake status between subjects with NCDs and subjects without NCDs. Significant differences in vegetable intake were found between subjects with hypertension or diabetes and subjects without hypertension or diabetes. In addition, more subjects with NCDs paid attention to eating breakfast daily. More subjects with hypertension, diabetes, and hyperlipidemia never ate out.

**Table 5:** The differences on dietary behavior between subjects with noncommunicable disease and subjects without noncommunicable disease.

		Hypertension		*P*	Diabetes		*P*	Hyperlipidemia		*P*	Angor pectoris		*P*
		Yes N (%)	No N (%*)*		Yes N *(*%*)*	No N *(*%*)*		Yes N *(*%*)*	No N *(*%*)*		Yes N (%*)*	No N *(*%*)*	
Salt-control	Yes	646 (77.2)	2126 (61.3)	<0.01	204 (71.1)	2568 (63.9)	<0.05	310 (73.1)	2462 (63.4)	<0.01	74 (71.8)	2698 (64.2)	0.119
	No	191 (22.8)	1343 (38.7)		83 (28.9)	1451 (36.1)		114 (26.9)	1420 (36.6)		29 (28.2)	1505 (35.8)	
Sugar-control	Yes	612 (73.0)	2068 (59.7)	<0.01	235 (81.9)	2445 (60.9)	<0.01	307 (72.2)	2373 (61.2)	<0.01	72 (69.9)	2608 (62.1)	0.122
	No	226 (27.0)	1398 (40.3)		52 (18.1)	1572 (39.1)		118 (27.8)	1506 (38.8)		31 (30.1)	1593 (37.9)	
Oil-control	Yes	650 (77.6)	2327 (67.1)	<0.01	216 (75.3)	2761 (68.7)	<0.05	331 (77.9)	2646 (68.1)	<0.01	79 (76.7)	2898 (68.9)	0.105
	No	188 (22.4)	1143 (32.9)		71 (24.7)	1260 (31.3)		94 (22.1)	1237 (31.9)		24 (23.3)	1307 (31.1)	
Food additives-control	Yes	573 (68.4)	1983 (57.3)	<0.01	208 (72.5)	2348 (58.5)	<0.01	296 (69.6)	2260 (58.4)	<0.01	77 (74.8)	2479 (59.1)	<0.01
	No	265 (31.6)	1477 (42.7)		79 (27.5)	1663 (41.5)		129 (30.4)	1613 (41.6)		26 (25.2)	1716 (40.9)	
Eat on time	Yes	683 (81.5)	2358 (68.1)	<0.01	240 (83.9)	2801 (69.1)	<0.01	340 (80.0)	2701 (69.7)	<0.01	86 (83.5)	2955 (70.4)	<0.01
	No	155 (18.5)	1106 (31.9)		46 (16.1)	1215 (30.3)		85 (20.0)	1176 (30.3)		17 (16.5)	1244 (29.6)	
Calorie-control	Yes	593 (70.8)	2132 (61.5)	<0.01	206 (71.8)	2519 (62.7)	<0.01	287 (67.4)	2438 (62.9)	0.072	72 (69.9)	2653 (63.2)	0.179
	No	244 (29.2)	1333 (38.5)		81 (28.2)	1496 (37.3)		139 (32.6)	1438 (37.1)		31 (30.1)	1546 (36.8)	
Nutrition balance	Yes	587 (70.0)	2359 (68.0)	0.282	203 (71.0)	2743 (68.3)	0.357	298 (70.0)	2648 (68.3)	0.510	68 (66.0)	2878 (68.5)	0.593
	No	251 (30.0)	1108 (32.9)		83 (29.0)	1276 (31.7)		128 (30.0)	1231 (31.7)		35 (34.0)	1324 (31.5)	
Drink adequate water	Yes	686 (81.9)	2613 (75.3)	<0.01	239 (83.6)	3060 (76.1)	<0.01	341 (80.2)	2958 (76.2)	0.070	85 (82.5)	3214 (76.5)	0.159
	No	152 (18.1)	855 (24.7)		47 (16.4)	960 (23.9)		84 (19.8)	923 (23.8)		18 (17.5)	989 (23.5)	
Refer to the nutrition label	Yes	334 (39.9)	1197 (34.5)	<0.01	118 (41.1)	1413 (35.2)	<0.05	168 (39.4)	1363 (35.1)	0.088	40 (38.8)	1491 (35.5)	0.532
	No	504 (60.1)	2269 (65.5)		169 (58.9)	2604 (64.8)		258 (60.6)	2515 (64.9)		63 (61.2)	2710 (64.5)	

*P* values were calculated using the Chi-square test.

**Table 6:** The differences on dietary intake status between subjects with noncommunicable disease and subjects without noncommunicable disease.

		Hypertension		*P*	Diabetes		*P*	Hyperlipidemia		*P*	Angor Pectoris		*P*
		Yes N (%)	No N (%)		Yes N (%)	No N (%)		Yes N (%)	No N (%)		Yes N (%)	No N (%)	
Fruit	<5 d/w	558 (67.1)	2426 (70.1)	0.111	186 (65.3)	2798 (69.8)	0.110	286 (67.8)	2689 (69.7)	0.436	65 (63.7)	2919 (69.6)	0.231
	≥5 d/w	273 (32.9)	1037 (29.8)		99 (34.7)	1211 (30.2)		136 (32.2)	1174 (30.3)		37 (36.3)	1273 (30.4)	
Vegetable	<5 d/w	242 (29.2)	1232 (35.6)	<0.01	80 (28.0)	1394 (34.9)	<0.05	126 (30.1)	1348 (34.9)	0.051	27 (26.2)	1447 (34.6)	0.092
	≥5 d/w	586 (70.8)	2225 (64.4)		206 (72.0)	2605 (65.1)		293 (69.9)	2518 (65.1)		76 (73.8)	2735 (65.4)	
Milk dairy	<5 d/w	606 (72.7)	2286 (66.5)	<0.01	201 (70.0)	2691 (67.5)	0.396	319 (75.8)	2573 (66.8)	<0.01	73 (72.3)	2819 (67.6)	0.335
	≥5 d/w	227 (27.3)	1153 (33.5)		86 (30.0)	1294 (32.5)		102 (24.2)	1278 (33.2)		28 (27.7)	1352 (32.4)	
Bea, tofu, soymilk	<5 d/w	688 (82.8)	2803 (81.2)	0.296	234 (82.4)	3257 (81.4)	0.752	352 (83.4)	3139 (81.3)	0.292	87 (85.3)	3404 (81.4)	0.367
	≥5 d/w	143 (17.2)	651 (18.8)		50 (17.6)	744 (18.6)		70 (16.6)	724 (18.7)		15 (14.7)	779 (18.6)	
Seaweed	<5 d/w	766 (92.1)	3155 (91.4)	0.627	258 (91.5)	3663 (91.6)	0.912	400 (95.2)	4521 (91.2)	<0.01	95 (92.2)	3826 (91.6)	1.000
	≥5 d/w	66 (7.9)	295 (8.6)		24 (8.5)	337 (8.4)		20 (4.8)	341 (8.8)		8 (7.8)	353 (8.4)	
Fish	<5 d/w	766 (92.7)	3233 (93.7)	0.346	256 (90.5)	3743 (93.7)	<0.05	402 (96.2)	3597 (93.2)	<0.05	93 (91.2)	3906 (93.5)	0.310
	≥5 d/w	60 (7.3)	219 (6.3)		27 (9.5)	252 (6.3)		16 (3.8)	263 (6.8)		9 (8.8)	270 (6.5)	
Meat	<5 d/w	688 (82.8)	2888 (83.7)	0.533	228 (80.0)	3348 (83.7)	0.116	347 (82.0)	3229 (83.7)	0.408	85 (82.5)	3491 (83.5)	0.788
	≥5 d/w	143 (17.2)	564 (16.3)		57 (20.0)	650 (16.3)		76 (18.0)	631 (16.3)		18 (17.5)	689 (16.5)	
Processed meat	<5 d/w	809 (97.4)	3312 (96.2)	0.119	280 (98.6)	3841 (96.2)	0.045	412 (97.4)	3709 (96.3)	0.274	102 (99.0)	4019 (96.3)	0.184
	≥5 d/w	22 (2.6)	132 (3.8)		4 (1.4)	150 (3.8)		11 (2.6)	143 (3.7)		1 (1.0)	153 (3.7)	
Instant noodle	<5 d/w	803 (96.7)	3338 (96.7)	1.000	275 (96.5)	3866 (96.7)	0.863	412 (97.2)	3729 (96.6)	0.668	96 (93.2)	4045 (96.8)	0.083
	≥5 d/w	27 (3.3)	115 (3.3)		10 (3.5)	132 (3.3)		12 (2.8)	130 (3.4)		7 (6.8)	135 (3.2)	
Breakfast	<5 d/w	142 (17.1)	923 (26.8)	<0.01	40 (14.1)	1025 (25.7)	<0.01	70 (16.6)	995 (25.8)	<0.01	15 (14.6)	1050 (25.2)	<0.05
	≥5 d/w	688 (82.9)	2521 (73.2)		244 (85.9)	2965 (74.3)		352 (83.4)	2857 (74.2)		88 (85.4)	3121 (74.8)	
Outdoor dining	<5 d/w	802 (96.2)	3049 (88.2)	<0.01	271 (94.8)	3580 (89.4)	<0.01	401 (94.4)	3450 (89.3)	<0.01	97 (94.2)	3754 (89.7)	0.185
	≥5 d/w	32 (3.8)	406 (11.8)		15 (5.2)	423 (10.6)		24 (5.6)	414 (10.7)		6 (5.8)	432 (10.3)	
Fast food	<5 d/w	822 (98.7)	3414 (98.6)	0.872	284 (99.0)	3952 (98.6)	0.797	419 (99.1)	3817 (98.5)	0.517	99 (96.1)	4137 (98.6)	0.057
	≥5 d/w	11 (1.3)	50 (1.4)		3 (3.0)	58 (1.4)		4 (0.9)	57 (1.5)		4 (3.9)	57 (1.4)	

*P* values were calculated using the Chi-square test.

## Discussion

To date, this is the first study on the current prevalence of NCDs and risk factor control in three Asian countries using a single study protocol. The prevalence rates of hypertension, diabetes, hyperlipidemia, and angina pectoris were highest in China and lowest in Japan. However, Chinese subjects presented greater awareness of risk factor control than Korean and Japanese subjects.

The lifestyle and diet of the Chinese, Korean, and Japanese people have changed substantially in recent decades, with increased fat and protein intake and a more sedentary lifestyle.^2^, ^3^, ^9^ A steady increase in NCDs has been observed because of these changes.^10^, ^11^ Many important international actions on NCDs have been initiated through international agencies, including the United Nations and WHO, during the past decade. WHO made important steps toward controlling major NCD risk factors by adopting the Framework Convention on Tobacco Control, Global Strategy on Diet, Physical Activity and Health, and Global Strategy to Reduce Harmful Use of Alcohol in 2003, 2004, and 2010, respectively.^12^ Over the past few decades, some significant achievements in risk factor modifications among Chinese, Koreans, and Japanese have been implemented. In Korea, Korean National Health Insurance Policy has provided most Koreans with medical examinations and health education every other year.^13^ In Japan, a 10-year national health promotion campaign, called Health Japan 21, was initiated by the Japanese government in 2000 for improving the nation's health.^14^ In this campaign, 59 indicators, such as diet, smoking, and diabetes, were established to monitor and improve risk factor management.^14^ In China, the national cancer prevention and control plan (2004–10) and a national chronic disease prevention and control plan have been implemented.^4^ In this report, about 82%, 48%, and 64% of Chinese, Koreans, and Japanese, respectively, presented good dietary behavior.

Although our dietary behavior quantification method is original to the present research, we discerned that greater attention is needed for modifying risk factors among subgroups of gender, age, and demographic status. We found that males are often less health consciousness compared with females, a finding which was consistent with those of previous studies.^13^, ^15^ In the Ansan study of Koreans, a significantly higher rate of awareness, treatment, and control of NCDs was observed in women than those in men.^13^ In the present research, subjects with hypertension, diabetes, or hyperlipidemia significantly improved their dietary behaviors and controlled salt, sugar, and oil intake compared with subjects without hypertension, diabetes, or hyperlipidemia. However, subjects with angina pectoris did not significantly improve their dietary behaviors compared with subjects without angina pectoris. Thus, health educators should target health education for patients with angina pectoris.

Our study has some limitations. This is a cross-sectional study than can only present the possible risk factors for health consciousness. Although we tried to standardize our surveys in three different regions as uniformly as possible, some procedures had to be modified according to the local research environment. The method of population recruitment and questionnaire survey had to be performed in ways that would best accommodate the local situation. For example, face-to-face interviews were conducted in China and Korea. However, a random sample of registered permanent residence and a mail survey were used in Japan. As a result, the response rate was significantly different among the three countries. The response rate was 55.6% in Japan. This rate is not low for a mail survey in Japan, but we should be careful about the relatively small population of younger respondents or working men. In addition, different understandings of salt, sugar, and oil control among the three countries that are based on cultural background may also introduce bias into the results. However, bias in the comparison of prevalence and risk control status among participants in the three countries is expected to be small because of the large sample size.

In conclusion, the current status of the prevalence of NCDs and trends in major modifiable risk factor control in China, Korea, and Japan reinforce the importance of prevention, detection, and treatment of risk factors in reducing the burden of NCDs on individuals and societies. Public efforts to introduce healthy lifestyle change and systematic NCD prevention programs are necessary to reduce the epidemic of NCDs in these three Asian countries.

## Conflicts of interest

None declared.
